# Mitochondria, Telomeres and Telomerase Subunits

**DOI:** 10.3389/fcell.2019.00274

**Published:** 2019-11-06

**Authors:** Qian Zheng, Jinliang Huang, Geng Wang

**Affiliations:** ^1^School of Life Sciences, Tsinghua University, Beijing, China; ^2^School of Life Sciences, Xiamen University, Xiamen, China

**Keywords:** mitochondria, telomere, telomerase, TERT, TERC, aging

## Abstract

Mitochondrial functions and telomere functions have mostly been studied independently. In recent years, it, however, has become clear that there are intimate links between mitochondria, telomeres, and telomerase subunits. Mitochondrial dysfunctions cause telomere attrition, while telomere damage leads to reprogramming of mitochondrial biosynthesis and mitochondrial dysfunctions, which has important implications in aging and diseases. In addition, evidence has accumulated that telomere-independent functions of telomerase also exist and that the protein component of telomerase TERT shuttles between the nucleus and mitochondria under oxidative stress. Our previously published data show that the RNA component of telomerase *TERC* is also imported into mitochondria, processed, and exported back to the cytosol. These data show a complex regulation network where telomeres, nuclear genome, and mitochondria are co-regulated by multi-localization and multi-function proteins and RNAs. This review summarizes the connections between mitochondria and telomeres, the mitochondrion-related functions of telomerase subunits, and how they play a role in crosstalk between mitochondria and the nucleus.

## Introduction

The aging field has seen a dramatic expansion in the last three decades with the discovery that it is controlled, at least to some extent, by evolutionally conserved pathways (Kenyon et al., 1993). Nine hallmarks of aging have since been postulated. The primary causal hallmarks are genomic instability, telomere damage, and epigenetic alterations, which are partially overlapped or intertwined with each other (Lopez-Otin et al., 2013). For example, telomere damage also leads to genomic instability (Fumagalli et al., 2012; Hewitt et al., 2012). Another hallmark is mitochondrial dysfunction, which is also closely related to genomic instability, as cells have two genomes: the nuclear genome and the mitochondrial genome. In addition, mitochondria are where the majority of intracellular reactive oxygen species (ROS) are produced (Wallace, 2005). The free radical theory of aging suggests that ROS cause oxidation damage in both mitochondrial DNA (mtDNA) and nuclear DNA that leads to accumulation of mutations and eventually aging (Harman, 1956). It is well known now that the theory is oversimplified and is only part of the story, as ROS may also activate compensatory pathways that may negate their deleterious effects (Yee et al., 2014; Wang and Hekimi, 2015).

Of all the attempts to decelerate aging, caloric restriction (CR) appears to be the only one that has an effect on most model species (Madeo et al., 2019). In the nucleus, CR upregulates DNA repair and ameliorates telomere erosion (Um et al., 2003; Wang C. et al., 2010; Szafranski and Mekhail, 2014). In mitochondria, it increases antioxidant functions and lowers ROS production (Lopez-Torres et al., 2002; Ruetenik and Barrientos, 2015). Subsequent studies showed that NAD^+^/sirtuin pathways play an important role in CR (Michan, 2014). SIRT1 and SIRT3 expression is upregulated by CR, and SIRT1 has been shown to be essential for CR mediated lifespan extension (Chen et al., 2008; Palacios et al., 2009; Mercken et al., 2014). In mammals, activation of SIRT1 stimulates mitochondrial biogenesis, and SIRT3 regulates CR-mediated metabolic responses by deacetylating proteins involved in mitochondrial functions (Hebert et al., 2013; Tang, 2016). In addition to NAD^+^/sirtuin pathways, AMPK-mTOR signaling has been shown to mediate the protective effects of CR in aging kidneys (Dong et al., 2017). However, both in yeast and mouse livers, CR and inhibition of mTOR pathway with rapamycin affect distinct metabolites, suggesting different mechanisms (Yu et al., 2015; Choi et al., 2017). Another pathway that is involved in the CR effects is the insulin/IGF-1 pathway. CR induces a reduction in both insulin and IGF-1 levels in most animals, and mice with disruption of the insulin signaling pathway have delayed aging biomarkers and increased lifespan (Argentino et al., 2005; Selman et al., 2008). However, it should be noted that the CR effect on IGF-1 levels is not universal. No effect of long-term CR on human IGF-1 levels has been observed (Fontana et al., 2008). These data suggest that the mechanism of CR is more complex than the few known signaling pathways. For mammals, exercise also has a generally beneficial effect against aging, and shares many molecular pathways with CR (Robinson et al., 2017; Escobar et al., 2019).

These discoveries together show that aging is regulated by multiple cellular processes that are interconnected and multiple organelles that communicate with each other. Stress–response pathways may be activated or upregulated during aging as a protective compensatory mechanism (Walker et al., 2006; Loerch et al., 2008). In this review, we will focus on mitochondrial dysfunction and telomere damage, the two aging hallmarks, and the two organelles where these processes occur: mitochondria and the nucleus.

Telomeres are structures with repetitive nucleotide sequences and particular binding proteins at the ends of eukaryotic chromosomes (Greider, 1991; de Lange, 2005). These structures are carefully maintained to safeguard genomic stability. In most human somatic cells that do not express telomerase, the enzyme capable of extending telomere DNA, progressive shortening of the DNA occurs with each cell division, which eventually leads to replicative senescence (Harley et al., 1990; Shay, 2016). In humans, telomere lengths in blood show a correlation with health and lifespan in individuals aged 60 years or older (Cawthon et al., 2003). However, it has also been shown that the percentage of short telomeres rather than the average telomere length is what matters in predicting lifespan (Vera et al., 2012). Telomerase is a holoenzyme that adds telomere repeat sequence to the 3′-end of telomeres (Shay and Wright, 2019). It is a ribonucleoprotein with an RNA subunit *TERC* and a reverse transcriptase enzymatic subunit TERT (Feng et al., 1995; Nakamura et al., 1997; Shay and Wright, 2019). Interestingly, human *TERC* is expressed in most normal human cells, but TERT is not detected in most, suggesting that *TERC* may have functions other than the template for telomere extension (Kim et al., 1994; Feng et al., 1995). It has now been reported that telomerase-independent functions of *TERC* do exist. They include inhibition of apoptosis in immune cells, protection of neuron cells from oxidative stress, and enhancement of cellular inflammatory responses (Gazzaniga and Blackburn, 2014; Eitan et al., 2016; Liu et al., 2019). TERT, however, is expressed in most cancer cells, some adult stem cells and some proliferating cells such as human T cells and B cells (Kim et al., 1994; Hiyama et al., 1995). It is also expressed in early embryos, but the expression is turned off in most somatic cells (Wright et al., 1996). In addition, expression in several non-dividing cells such as neurons and cardiac myocytes has been reported and telomere-independent roles in protecting these cells from oxidative damage and other stresses have been proposed (Kang et al., 2004; Richardson et al., 2012; Iannilli et al., 2013). The expression pattern of TERT is also different in rodents from that in humans. For example, rodents continue to express TERT in several tissues throughout life (Golubovskaya et al., 1997). These different patterns may have implication in cancer development in long-lived mammals such as humans. The sub-nuclear localization of TERT protein, the localization of the *TERT* gene, and the 3D interaction of telomeres with other sections of the genome and the nuclear envelope all appear to play a role in fine tuning the timing and functions of telomerase and telomeres (Wood et al., 2014, 2015; Robin et al., 2015).

Deletion of either *TERT* or *TERC* in mice results in a complete loss of telomerase activity, shortens the life span of late generation mice, reduces their stress-responding capacity, and increases spontaneous malignancies in highly proliferative tissues as a consequence of chromosomal instability (Rudolph et al., 1999; Yuan et al., 1999). The appearance of these tumors is consistent with the finding that telomerase is not required for oncogenic transformation (Blasco et al., 1997). However, these late-generation telomerase-deficient mice are more resistant to multi-stage skin carcinogenesis, suggesting that for cell types that are less sensitive to chromosomal instability induced by telomere loss, telomerase activity may still be a target for cancer therapy (Gonzalez-Suarez et al., 2000). TERT overexpression has been shown to increase the maximum life span of mice up to 10% and reduce the incidence of certain age-related degenerative diseases (Gonzalez-Suarez et al., 2001, 2005). However, mice overexpressing TERT are also more susceptible to both induced and spontaneous tumorigenesis, as a result of TERT driving cell proliferation (Gonzalez-Suarez et al., 2001, 2005). It should be noted that *TERC* expression is limiting for telomere maintenance, as *terc^+/–^* heterozygotes but not *tert^+/–^* heterozygotes are deficient in telomere elongation (Chiang et al., 2004). Overexpressing TERT but not *TERC* may not increase the total telomerase activity, as the overexpressed TERT may not have *TERC* to form telomerase with. Whether the effect of TERT overexpression is telomerase dependent remains to be examined. Attempts have been made by overexpressing TERT in the *terc^–/–^* background (Cayuela et al., 2005). However, the results are not direct answers to the question, because *TERC* deletion itself has telomerase-independent effects on cells and animals.

Recent discoveries have also shown that telomere maintenance is not simply regulated by nuclear events. Nuclear processes are also tightly regulated by signals coming from outside of the nucleus. One of the best examples is the forward and backward trafficking of proteins and metabolites, and signaling between mitochondria and the nucleus. These co-regulations could be temporal or under specific conditions. For example, nuclear localization of mitochondrial TCA cycle enzymes has been shown to be a critical step in mammalian zygotic genome activation (Nagaraj et al., 2017). Telomere maintenance, subcellular localization of telomerase subunits, and mitochondrial functions also share some intimate links, which are reviewed in the following sections.

## Mitochondrial Dysfunctions Induce Telomere Damage

**FIGURE 1:**
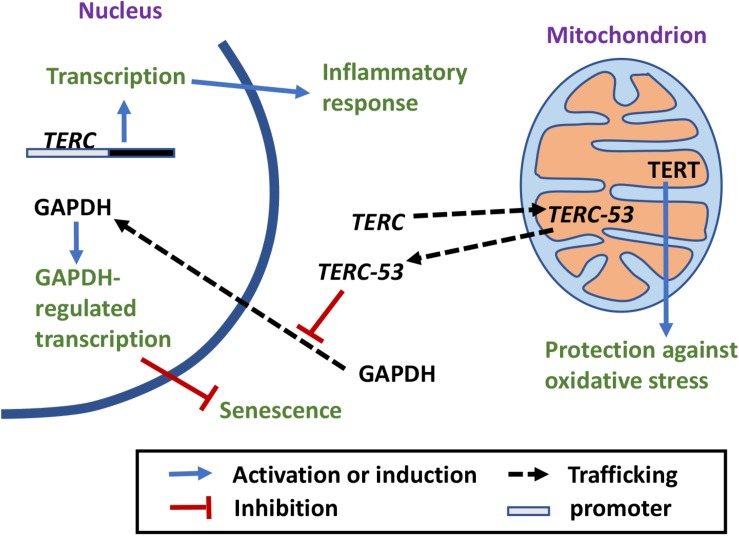
The crosstalk between mitochondria and telomeres. Mitochondrial defects increase ROS release, which has a damaging effect on telomeres (Sohal et al., 1994; von Zglinicki et al., 1995, 2000; Korshunov et al., 1997; Oikawa and Kawanishi, 1999; Liu et al., 2002; Saretzki et al., 2003). Antioxidant treatment ameliorates the negative effect (Serra et al., 2003; Stauffer et al., 2018). Telomere damages lead to mitochondrial biosynthesis reprogramming and mitochondrial dysfunction through different signaling pathways (Simpson and Russell, 1998; Biswas et al., 2005; Iwabu et al., 2010; Passos et al., 2010; Guo et al., 2011; Sahin et al., 2011; Scarpulla, 2011; Correia-Melo et al., 2016).

Mitochondria are the power plants of eukaryotic cells and also the main site of ROS production. A significant portion of the electrons that pass from reduced substrates to oxygen in the mitochondrial respiratory chain leak to form superoxide (Raha and Robinson, 2000). Superoxide, although relatively unreactive itself, is the parent of a number of damaging ROS (Beckman and Koppenol, 1996). Whether a cell experiences oxidative stress is dependent on the balance between ROS production, their removal, and the activities of the damage repair pathways. Changes that occur to mitochondrial activity may significantly increase ROS levels and cause oxidative stress (Korshunov et al., 1997; Figure 1). High oxidative stress leads to breakage of single and double strand DNA (Sohal et al., 1994). It also increases the rate of telomere shortening, as telomeres are rich in guanine and more susceptible to oxidative damage (von Zglinicki et al., 1995; Oikawa and Kawanishi, 1999; Figure 1). H_2_O_2_ plus Cu^2+^ induces 8-oxo-7,8-dihydro-2′-deoxyguanosine (8-oxodG) formation more efficiently in telomere sequences than in non-telomere sequences, suggesting that the sequence context may also play a role in the oxidation (Oikawa and Kawanishi, 1999). Treatment of human fibroblasts with hydrogen peroxide leads to an acceleration of telomere shortening in these proliferating cells, while a reduction of intracellular peroxide activities by alpha-phenyl-*t*-butyl-nitrone decreases the shortening rate (von Zglinicki et al., 2000; Figure 1). Another antioxidant MitoQ that is targeted specifically to mitochondria also shows a similar decelerating effect on telomere shortening, while mitochondrial depolarization in mouse embryos with FCCP [carbonyl cyanide-4-(trifluoromethoxy)phenylhydrazone] treatment leads to an increase in ROS production and telomere shortening, suggesting a connection between mitochondrial dysfunctions and telomere shortening (Liu et al., 2002; Saretzki et al., 2003).

Patients with primary mitochondrial disorders (such as diseases caused by mutations in mitochondrial genome) and secondary dysfunctions (such as metabolic diseases, neurodegenerative diseases, and others) also have shorter telomeres compared to healthy controls (Gonzales-Ebsen et al., 2017). Studies done on highly stressed women and people with major depressive disorders also show a correlation between mitochondrial dysfunction, oxidative stress, and telomere shortening (Epel et al., 2004; Simon et al., 2006; Lindqvist et al., 2015).

Cells, however, possess their own machineries for ROS detoxification. For example, superoxide is scavenged by cytochrome c, and superoxide dismutases in the matrix (MnSOD), cytoplasm (Cu/Zn SOD), and the extracellular space (EC-SOD) (Raha and Robinson, 2000; Serra et al., 2003). Mice with higher superoxide dismutase activity have longer telomeres (Stauffer et al., 2018). Induction of an extracellular superoxide dismutase in the presences of mitochondrial dysfunctions has been shown to mitigate the effects of oxidation stress and slow telomere shortening (Serra et al., 2003).

## Telomere Damage Leads to Mitochondrial Biosynthesis Reprogramming and Mitochondrial Dysfunctions

Among all the organelles, mitochondria probably are the most sensitive to environmental cues, and can quickly change their functional state and energy output to meet the needs of the cells. The number of mitochondria in a cell is controlled by the balance between mitochondrial biosynthesis and mitophagy. Mitochondrial biosynthesis itself involves multiple organelles and is the concerted result of mtDNA replication, transcription, and mitochondrial RNA (mtRNA) translation within mitochondria, and the import of macromolecules (such as proteins and RNAs) and smaller molecules (such as lipids) into mitochondria. Mitochondrial biosynthesis is regulated by the PGC-1 family of coactivators that consist of PGC-1α, PGC-1β, and PRC (Scarpulla, 2011). Expression of these coactivators is modulated by extracellular signals that control metabolism, differentiation, or cell growth (Scarpulla, 2011). Post-translational modification by energy sensors such as AMPK and SIRT1 has also been shown to modulate their activities (Iwabu et al., 2010; Scarpulla, 2011). In addition, mitochondrial biosynthesis responds to mitochondrial functional states. Low mitochondrial membrane potential in damaged mitochondria affects mitochondrial intake of cytosolic Ca^2+^, which changes cytosolic Ca^2+^ levels and Ca^2+^ signaling in the cytosol, consequently changing the gene expression pattern in the nucleus and promoting mitochondrial biosynthesis (Simpson and Russell, 1998; Biswas et al., 2005).

In 2009, a study in mice showed that short telomeres reduce mitochondrial membrane hyperpolarization and Ca^2+^ influx in β-cells (Guo et al., 2011; Figure 1). A possible mechanism for short telomeres to affect mitochondrial functions is through DNA damage responses (DDRs). Overexpression of a dominant-negative telomere-binding protein TRF2 induces DDR, which activates p21, and in turn induces mitochondrial dysfunction and increases ROS production through GADD45-MAPK14-GRB2-TGFBR2-TGFβ signaling cascade. These ROS induce more DNA damage, maintaining an ongoing DDR. This vicious loop locks the cells in a deep senescence state (Passos et al., 2010; Figure 1). In a 2011 study, p53 and PGC coactivators were identified as the master regulators that link telomere dysfunction and mitochondrial compromise (Sahin et al., 2011). Telomere dysfunction activates p53 and the activated p53 binds *PGC-1*α and *PGC-1*β promoters, suppressing their expression and in turn mitochondrial biosynthesis (Sahin et al., 2011; Figure 1). More importantly, forced expression of PGC-1α or deletion of p53 in the setting of telomere dysfunction restores mitochondrial respiration (Sahin et al., 2011). In addition, the ATM, Akt, and mTORC1 phosphorylation cascade has been shown to integrate signals from DDR to PCG-1β-dependent mitochondrial biogenesis, contributing to DDR activation by ROS, which eventually leads to cellular senescence (Correia-Melo et al., 2016). mTORC1 inhibition or *PCG-1*β deletion reduces mitochondrial content and prevents senescence in aging mouse livers (Correia-Melo et al., 2016). It appears that telomere damage or nuclear DNA damage in general could affect mitochondrial functions and mitochondrial biosynthesis through multiple signaling pathways.

## Tert Shuttles Between the Nucleus and Mitochondria Under Oxidative Stress

**FIGURE 2:**
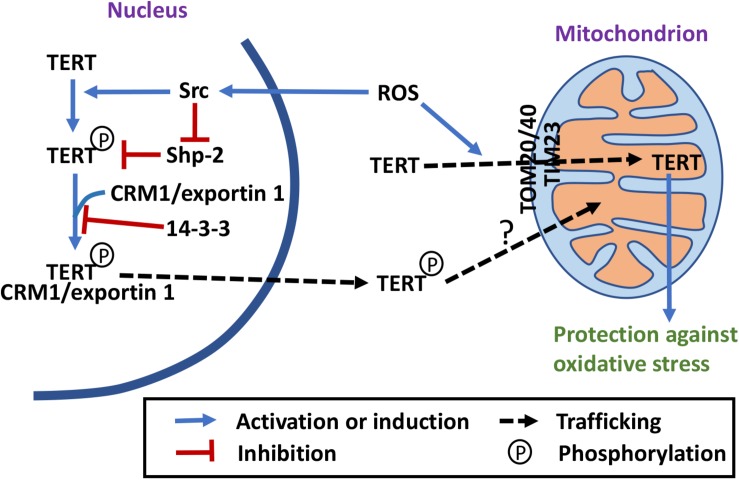
Telomerase-independent trafficking and functions of TERT. Under stress conditions, TERT is exported out of the nucleus and imported into mitochondria, where it may have a protective role (Seimiya et al., 2000; Haendeler et al., 2003, 2004, 2009; Santos et al., 2004; Yan et al., 2004; Jakob et al., 2008; Maida et al., 2009; Kovalenko et al., 2010; Sharma et al., 2012; Singhapol et al., 2013; Miwa et al., 2016; Green et al., 2019). Phosphorylation by Src and dephosphorylation by Shp-2 regulate TERT nuclear export (Haendeler et al., 2003; Jakob et al., 2008). The NES motif of TERT interacts with the nuclear export receptor CRM1/exportin, which is inhibited by 14-3-3 binding (Seimiya et al., 2000; Haendeler et al., 2003). Import of TERT into mitochondrial depends on a N-terminal MTS, TOM20 and TOM40 translocases at the outer membrane and TIM23 translocase at the inner membrane (Haendeler et al., 2009; Kovalenko et al., 2010; Sharma et al., 2012; Green et al., 2019).

Recent discoveries have also shown that there are extra-nuclear pools of telomerase protein TERT. The functions of TERT outside of the nucleus were originally defined as telomere independent functions. It, however, has become abundantly clear that these functions also play important roles in telomere maintenance, genomic stability, and the crosstalk between the nucleus and mitochondria. In 2004, an immunohistochemical study showed that in TERT-positive cells, TERT could not only be detected in the nucleus, but also occasionally in the cytoplasm (Yan et al., 2004). It was later shown that in fully differentiated neurons, cytoplasmic TERT interacts with the messenger RNA (mRNA) of the cyclin kinase inhibitor p15INK4B, suppressing its translation (Iannilli et al., 2013). Under stress conditions, the mRNA is released and p15NK4B protein is translated as a pro-survival response (Iannilli et al., 2013). In endothelial cells, continuous cultivation results in an increase of endogenous ROS accompanied by mtDNA damage and export of TERT from the nucleus to the cytoplasm (Haendeler et al., 2004; Figure 2). Treating the cells with the antioxidant *N*-acetyl cysteine (NAC) decreases the nuclear export and delays the onset of replicative senescence (Haendeler et al., 2004). A nuclear export signal (NES) motif that interacts with nuclear export receptor CRM1/exportin has been identified at the C-terminus of TERT (Seimiya et al., 2000; Haendeler et al., 2003; Figure 2). The export requires an active nuclear Ran GTPase and is inhibited by binding of 14-3-3 proteins to TERT (Seimiya et al., 2000; Haendeler et al., 2003). Under oxidative stress, TERT nuclear export is regulated by phosphorylation. Src kinase phosphorylates TERT on tyrosine 707, which induces TERT nuclear export and is reversed by a protein tyrosine phosphatase Shp-2 (Haendeler et al., 2003; Jakob et al., 2008; Figure 2). A mitochondrial localization sequence at the N-terminus of TERT has also been identified (Santos et al., 2004; Haendeler et al., 2009). In Hela cells that express endogenous telomerase and in NHF primary fibroblasts, an exogenous TERT–EGFP has been detected in mitochondria (Santos et al., 2004). NHF fibroblasts expressing TERT also display higher level of mtDNA damage (Santos et al., 2004). However, other studies have shown that overexpression of TERT has a protective effect on mtDNA integrity under oxidative stress (Haendeler et al., 2004, 2009). It should be noted that the nuclear export of endogenous TERT was detected both in the endothelial cells and HEK293 cells, but the mitochondrial localization in these studies was examined with overexpression of an exogenous TERT. The discrepancy between the effects of TERT overexpression on mtDNA integrity could be the results of different TERT overexpression levels or different growth conditions of the cells. The sub-mitochondrial localization of TERT was later shown to be in the mitochondrial matrix with another ectopically overexpressed TERT (Figure 2). Interaction of TERT with TOM20 and TOM40 at the mitochondrial outer membrane and TIM23 at the inner membrane has also been observed, suggesting an active mitochondrial import mechanism (Haendeler et al., 2009; Figure 2). In the mitochondrial matrix, TERT binds to mtDNA at ND1 and ND2 coding regions, and protects mtDNA against ethidium-bromide treatment (Haendeler et al., 2009). In addition, it has been shown that mitochondrion-targeted TERT has the most protective effect on H_2_O_2_-induced apoptosis (Haendeler et al., 2009). In a 2010 study, mutant TERT proteins that are disrupted in the NES but retain the enzymatic activity were used to better understand the biological effects of TERT shuttling (Kovalenko et al., 2010). The mutant failed to immortalize cells compared to its wild-type counterpart. Overexpression of the mutant also has deleterious effect on mitochondrial functions and resulted in higher levels of mitochondrial ROS, damage in telomeric and extratelomeric DNA, and premature senescence in fibroblasts (Kovalenko et al., 2010). Even though the mutant is catalytically active in a cell-free extract, it failed to extend telomeres in the cell (Kovalenko et al., 2010). It is possible that the NES region is somehow involved in telomere maintenance in the cell. Another possibility is that the mitochondrial dysfunction caused by the mutant expression has a negative effect on telomere that outweighs the positive effect of an active telomerase. However, it remains to be answered why expression of the nucleus-only mutant in telomerase negative cells has a negative effect on mitochondrial functions. It is possible that nuclear gene expression pattern is affected by the damage in telomeric and extratelomeric DNA caused by the mutant expression, hence affecting mitochondrial function.

In 2009, the RNA component of mtRNA processing endoribonuclease (RMRP) was shown to interact with TERT and form a complex with RNA polymerase activity *in vitro* (Maida et al., 2009). However, whether this enzymatic activity exists in mitochondria *in vivo* remains to be examined. In 2012, endogenous TERT was detected in mitochondria in HEK293 cells (Sharma et al., 2012; Figure 2). In the same study, an ectopically overexpressed TERT was shown to interact with mitochondrial tRNAs, *RMRP*, and *5.8S* rRNA (Sharma et al., 2012). It was also shown that in a cell-free extract, TERT functions as a *TERC*-independent reverse transcriptase, using mitochondrial tRNAs as templates (Sharma et al., 2012). However, whether the protein is involved in synthesis of mtDNA *in vivo* remains unanswered. In an attempt to understand the biological function of TERT in the cell, fibroblasts untransfected or stably expressing WT or mutant TERT [with mutations in the mitochondrial targeting sequence (MTS)] that is catalytically active in the nucleus and proficient in cellular immortalization, but unable to enter mitochondria were compared (Sharma et al., 2012; Green et al., 2019). The results, however, only suggest that mitochondrial localization of an ectopically overexpressed TERT positively affects mitochondrial function and inhibits H_2_O_2_-induced autophagy, but not that lack of TERT in the mitochondria negatively affects mitochondrial function and activates H_2_O_2_-induced autophagy.

In cancer Hela and MCF7 cells, export of endogenous TERT from the nucleus and import of TERT into mitochondria has also been observed after H_2_O_2_ treatment (Singhapol et al., 2013). The nuclear TERT exclusion lasts up to several days after a single bolus dose of H_2_O_2_, and positive correlation of the exclusion and the protection effect against nuclear DNA damage has been observed (Singhapol et al., 2013). Overexpression of the mitochondrion-localized TERT, but not the nucleus-localized TERT, has a protective effect on nuclear DNA damage and apoptosis after H_2_O_2_ treatment (Singhapol et al., 2013). These results suggest that cancer cells use nuclear exclusion and mitochondrial targeting of TERT to protect themselves from nuclear DNA damage and apoptosis.

In mouse brains, TERT expression decreases with age while the release of ROS from mitochondria increases (Miwa et al., 2016). Dietary restriction (DR) leads to an accumulation of mitochondrial TERT that correlates to decreased ROS production and improved cognition (Miwa et al., 2016). Rapamycin treatment has a similar effect on mitochondrial TERT level and ROS release as DR does. The beneficial effect of rapamycin on mitochondrial functions is absent in the brains of *tert^–/–^* mice (Miwa et al., 2016). Rapamycin-induced shuttling of TERT out of the nucleus is dependent on Src kinase, and a Src kinase inhibitor bosutinib blocks the rapamycin effect of ROS reduction (Miwa et al., 2016). These results again suggest that mitochondrial TERT has a protective effect on mitochondrial functions in the mouse brain and in cellular models. Even though it was not specified in this study what kind of brain cells this process occurs, a previous paper by the same group showed that TERT is expressed in mouse and human neurons and microglia cells, but not in astrocytes (Spilsbury et al., 2015).

In conclusion, mitochondrial localization of TERT is a cell type-specific event that protects the cells from oxidative stress. What is the exact function of mitochondrion-localized TERT within the mitochondrial matrix, however, remains to be elucidated. Whether the mitochondrial pool of TERT is from the nucleus also remains to be examined. Src activity has been shown to be involved in the nuclear export of TERT, but in mitochondria, it has been proposed to be involved in its degradation (Haendeler et al., 2003; Buchner et al., 2010). These data suggest that TERT exported from the nucleus is mostly phosphorylated but TERT imported into mitochondria is probably not. That the mutations in NES and those in MTS have different effects on TERT’s capacity to immortalize cells also suggest that TERT mitochondrial import may not be strictly downstream of TERT nuclear export (Kovalenko et al., 2010; Sharma et al., 2012; Green et al., 2019). More studies are needed to understand this part of trafficking.

## Mitochondrial Localization and Processing of Terc

Mitochondria import not only nucleus-encoded proteins but also RNAs from the cytosol. These RNAs include tRNAs and other non-coding RNAs (Mercer et al., 2011). Studies on mtRNA import started as early as 1967, and the first report was about nucleus-encoded tRNAs in the mitochondria of *Tetrahymena pyriformis* (Suyama, 1967). In the following years, more evidence accumulated. In 1988, it was shown in bean *Phaseolus vulgaris*, four mitochondrial *tRNA*(*Leu*) are encoded in the nuclear genome, and that these tRNAs are competent in protein translation even though they have different post-transcriptional modification from their cytosolic counterparts (Marechal-Drouard et al., 1988). It was later discovered that mitochondrial tRNA import also occurs in both *Trypanosoma brucei* and *Saccharomyces cerevisiae* (Mottram et al., 1991; Rinehart et al., 2005). In 2008, studies on mammals showed that both rat and human mitochondria also import tRNAs by a mechanism distinct from protein import (Rubio et al., 2008). In 2009, microRNAs that are potentially involved in apoptosis regulation were identified in rat liver mitochondria (Kren et al., 2009). In another study, microRNAs were shown to directly enhance mitochondrial translation during muscle differentiation (Zhang et al., 2014).

**FIGURE 3:**
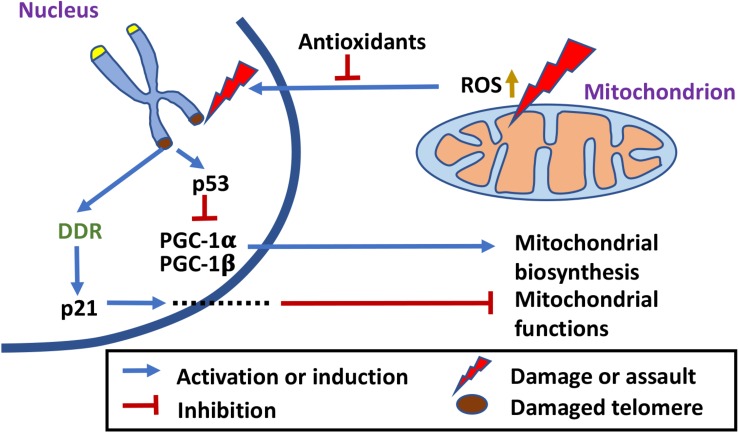
Telomerase-independent trafficking and functions of *TERC*. *TERC* is imported into mitochondria, processed to *TERC-53*, and then exported back to the cytosol where it inhibits nuclear translocation of GAPDH (Luhtala and Parker, 2010; Hillwig et al., 2011; Liu et al., 2017; Cheng et al., 2018; Huang et al., 2018). Cytosolic *TERC-53* relays the signal of mitochondrial defects to the nucleus and is involved in cellular senescence and organismal aging. Nuclear *TERC* can also bind directly to the promoter regions and upregulate transcription of genes that are involved in inflammatory response (Liu et al., 2019).

A mitochondrial intermembrane space (IMS) protein PNPASE was later shown to be directly involved in regulating mtRNA import and a small stem-loop structure on the imported RNAs has been identified as a possible import signal (Wang G. et al., 2010, 2012). *TERC* also possesses such a stem loop structure, and was shown to be imported into mitochondria, processed within mitochondria to a smaller product *TERC-53*, and then exported back to the cytosol (Cheng et al., 2018; Figure 3). A previous study on mitochondrial TERT localization also explored the possibility of mitochondrial localization of *TERC* (Sharma et al., 2012). However, only RT-PCR was used to examine the mitochondrial fraction and no product was obtained. We have also done the RT-PCR and only primers that anneal to the processed *TERC* fragment could amplify the RNA (Cheng et al., 2018). The import of *TERC* into mitochondria is also regulated by mammalian PNPASE that unlike its protozoan counterparts does not possess a ribonuclease activity (Liu et al., 2017; Cheng et al., 2018). Instead, processing of *TERC* is performed by a mitochondrial IMS protein RNASET2 (Liu et al., 2017; Cheng et al., 2018; Huang et al., 2018). RNASET2 is a protein of multi-localization. It has been shown to be also localized in the lysosomes (Luhtala and Parker, 2010). Even though the RNASET2 proteins in most other species have a preference for lower pH, mammalian RNASET2 and a RNASET2 enzyme in *Arabidopsis thaliana* RNS2 that is localized to endoplasmic reticulum (ER) have very low activities at lysosomal pH of 5.0, indicating adaptation of the protein activities to their localizations (Luhtala and Parker, 2010; Hillwig et al., 2011; Liu et al., 2017).

Interestingly, the majority of *TERC-53* has been detected in the cytosol instead of mitochondria, and the cytosolic levels of *TERC-53* are regulated by mitochondrial functions but have no direct effect on mitochondria (Cheng et al., 2018; Figure 3). The following studies showed that *TERC-53* functions as a signaling molecule, relating the functional states of mitochondria to the nucleus, possibly by inhibiting GAPDH nuclear translocalization (Zheng et al., 2019). *TERC-53* itself does not affect and is independent of telomerase activity (Zheng et al., 2019). The whole process of *TERC-53* production and function has been shown to be involved in cellular senescence and organismal aging (Zheng et al., 2019; Figure 3).

Unlike TERT that is not expressed in most tissues, human *TERC* is expressed in most tissues (Kim et al., 1994; Feng et al., 1995). Its functions are most likely broader than those of TERT. Like TERT, whether there is a specific function of *TERC* within mitochondria and if so, what the function is, however, remains to be fully elucidated. Telomerase-independent functions of *TERC* have been reported before. Overexpression of telomerase-inactive *TERC* protects stimulated CD4 T cells from dexamethasone-induced apoptosis, while *TERC* knockdown but not TERT knockdown induces apoptosis without causing telomere shortening or DDR (Gazzaniga and Blackburn, 2014). In motor neuron cells, overexpression of an alternative *TERC* (*alTERC*) protects the cells from oxidative stresses without altering TERT protein level or telomerase activity (Eitan et al., 2016). These observations still await mechanistic characterization and whether mitochondria are directly involved or not remains to be examined. A recent study has revealed another telomerase-independent function of *TERC* and a new mechanism by which *TERC* promotes an inflammatory response (Liu et al., 2019). However, in this case, mitochondria are not directly involved. *TERC* was shown to enhance the expression and release of inflammatory cytokines by directly binding to the promoters of the genes encoding proteins that are involved in activation of the NF-κB pathway (Liu et al., 2019; Figure 3). These results again suggest that *TERC* has different functions at different locations within the cell and that there are different mechanisms of telomerase-independent functions.

## Concluding Remarks

It has now become clear that the signaling between mitochondria and the nucleus is a complex network of events with proteins and RNAs as additional signaling molecules. There are lessons to be learned from the studies on mitochondria, telomeres, telomerase subunits, and aging in general. Aging does not start from a single trigger. It is a process involving multiple organelles within a cell and multiple organs in multicellular organisms. There are big variations between cell types and species, and changes are both spatial and temporal, as shown by the expression and trafficking of TERT and *TERC*. Multi-localization of biological molecules could be involved in co-regulation of processes in different cellular compartments. Similar proteins in different species do not necessarily have similar functions, and same biological molecules in different cellular compartments also do not necessarily have similar functions. Fine tuning of the complex regulation networks plays big roles in normal aging of multicellular organisms, and this fine tuning includes both positive and negative feedbacks that are not necessarily linear events as cells and animals age.

## Author Contributions

QZ, JH, and GW contributed in writing and editing of the manuscript.

## Conflict of Interest

The authors declare that the research was conducted in the absence of any commercial or financial relationships that could be construed as a potential conflict of interest.
